# Biochanin A Suppresses Tumor Progression and PD-L1 Expression via Inhibiting ZEB1 Expression in Colorectal Cancer

**DOI:** 10.1155/2022/3224373

**Published:** 2022-02-22

**Authors:** Junying Xu, Xuejing Yang, Jiadong Pan, Honghong Fan, Jie Mei, Dong Hua

**Affiliations:** ^1^Department of Oncology, The Affiliated Wuxi People's Hospital of Nanjing Medical University, Wuxi 214023, Jiangsu, China; ^2^Department of Gastroenterology, The Affiliated Wuxi People's Hospital of Nanjing Medical University, Wuxi 214023, Jiangsu, China

## Abstract

**Objective:**

To investigate the regulatory effect of ZEB1 on PD-L1 expression and the pharmacodynamic effects of Biochanin A on the malignant biological behaviors of colorectal cancer (CRC).

**Methods:**

The correlation between epithelial-mesenchymal transition (EMT) score and features of the tumor microenvironment (TME) was investigated using the Cancer Genome Atlas (TCGA) dataset. The correlation between ZEB1 and PD-L1 expression was validated using immunohistochemistry (IHC) staining, and the regulatory effect of ZEB1 on PD-L1 expression was explored by in vitro assays. Moreover, the pharmacodynamic effects of Biochanin A on ZEB1 and PD-L1 expression, as well as malignant biological behaviors of CRC cells, were evaluated by in vitro and in vivo assays.

**Results:**

EMT score was positively correlated with a majority of immunostimulators, immune checkpoints, activities of antitumor immunity cycles, and infiltration levels of most immune cells in the TCGA dataset. In addition, ZEB1 was correlated with and positively regulated PD-L1 expression in CRC. Besides, Biochanin A, an inhibitor for the ZEB1/PD-L1 axis, notably inhibited ZEB1-mediated aggressiveness and PD-L1 expression of CRC cells. Moreover, Biochanin A also exerted a tumor-inhibitory role in vivo in the CRC mouse model.

**Conclusion:**

Overall, we found that ZEB1 is a main regulator of PD-L1 expression in CRC. In addition, we also identified Biochanin A as a novel inhibitor for the ZEB1/PD-L1 axis, which could inhibit tumor progression and immune escape.

## 1. Introduction

Colorectal cancer (CRC) is one of the most widespread digestive cancers, ranking third in both morbidity and mortality [1]. Based on the latest cancer statistics, approximately 149,550 cases of CRC will arise, and 52,980 CRC-associated deaths will occur in the United States in 2021 [1]. In recent years, the therapeutic strategies for CRC, including surgery, chemotherapy, radiotherapy, and immunotherapy, have been rapidly and largely developed. Thus, the 5-year overall survival (OS) rate for CRC patients has reached 65% and even 90% in early-stage CRC patients [2, 3]. Nonetheless, patients with advanced-stage CRC still face lethal clinical outcomes due to uncontrollable metastasis and other complications. Thus, it is urgent to explore the mechanisms underlying oncogenesis and search for more promising strategies for CRC.

Epithelial-mesenchymal transition (EMT) is a specific biological process in which epithelial cells transform into cells with a mesenchymal phenotype. EMT acts as a significant role in multiple physiological and pathological conditions, such as embryonic development, chronic inflammation, tissue reconstruction, tissue fibrosis, and malignant tumor progression [4]. Through the EMT process, epithelial cells lose cell polarity, the connection with basement membrane, and other epithelial features but obtain the mesenchymal features, such as high invasive capacity, antiapoptosis, and ability to degrade extracellular matrix [5]. Emerging studies reveal that EMT can significantly promote the progression and immune evasion of malignant tumors [6–8]. For example, kallikrein-related peptidase 8 (KLK8) promotes the growth and metastasis of CRC via activating the EMT process [9]. Therefore, the exploration of therapeutic strategies targeting the EMT process may be a critical breakthrough point to control the progression of CRC.

In the current study, we explored the correlated pattern of EMT-related gene expression and features of the tumor microenvironment (TME) utilizing the data from the Cancer Genome Atlas (TCGA) dataset. Next, the correlation and regulatory axis of ZEB1 and PD-L1 were investigated. Besides, we validated the tumor-suppressive role of Biochanin A in CRC in vitro and in vivo, which could suppress the ZEB1-mediated progression and PD-L1 expression. Overall, our findings uncover the critical role of ZEB1 in promoting tumor progression and identify a novel inhibitor Biochanin A to block the ZEB1/PD-L1 axis.

## 2. Materials and Methods

### 2.1. Public Data Acquisition and Bioinformatics Analysis

The normalized RNA sequencing (RNA-seq) for CRC from the TCGA dataset was obtained from the UCSC Xena portal (https://xenabrowser.net/datapages/). Next, the immunobiological correlations of EMT enrichment score in CRC were evaluated, which was calculated by single-sample gene set enrichment analysis (ssGSEA) [10] referring to a penal of EMT-associated genes belonging to gene sets “HALLMARK-EPITHELIAL-MESENCHYMAL-TRANSITION” (*n* = 200) in the Molecular Signatures Database (MSigDB) [11].

Given that the tumor tissues which were subjected to RNA-seq included tumor cells and other cells, such as immune cells, we assessed immunological characteristics of the TME for each patient from the TGCA dataset. First, the information of 122 immunomodulators and well-known effector genes of TIICs was also collected from previous studies [12]. Besides, ESTIMATE was used to estimate the Tumor Purity, ESTIMATE Score, Immune Score, and Stromal Score [13]. In addition, five independent algorithms, including TIMER [14], EPIC [15], MCP-counter [16], quanTIseq [17] and TISIDB [18], were utilized to compute the relative abundance of tumor-infiltrating immune cells (TIICs) comprehensively. Furthermore, considering that each stage of the cancer immune cycle plays a crucial role in reflecting the anticancer immune response and deciding the destiny of tumor cells, we next calculated the activities of each stage by ssGSEA according to the expression level of stage-specific signatures [10]. To investigate the immunobiological role of EMT in CRC, we divided the patients into the high and low EMT score groups at the 50% cutoff criterion and then compared the difference of immunological features of TME between the high and low EMT subtypes.

### 2.2. Reagents and Antibodies

Biochanin A (Cat. B106472) was purchased from Aladdin (Shanghai, China). Ready-to-use PD-L1 antibody (rabbit mAb, Cat. GT2280) was purchased from GeneTech (Shanghai, China). Antibodies targeting ZEB1 (rabbit pAb, Cat. 21544-1-AP), PD-L1 (mouse mAb, Cat. 66248-1-Ig), E-cadherin (rabbit pAb, Cat. 20874-1-AP), N-cadherin (rabbit pAb, Cat. 22018-1-AP), and GAPDH (mouse mAb, Cat. 60004-1-Ig) were purchased from ProteinTech (Wuhan, China).

### 2.3. Tissue Microarray

The CRC tissue microarray (TMA) section (Cat. HColA160Bc01) was obtained from Outdo BioTech (Shanghai, China). This TMA contained 80 tumor and their paired paratumor samples. The relevant medical record of clinic-pathological features for each sample was obtained from Outdo BioTech. Ethical approval for the study of the TMA was granted by the Clinical Research Ethics Committee, Outdo BioTech (Shanghai, China).

### 2.4. Immunohistochemistry

Immunohistochemistry (IHC) staining was performed on the section according to the standard steps. Briefly, the TMA was deparaffinized at 55°C for 30 min and then washed with xylene for three 5 min. The section was rehydrated by successive washes in 100%, 90%, and 70% graded ethanol. Hydrogen peroxidase (0.3%, ZSGB-Bio, Beijing, China) was used to block endogenous peroxidase activity for 20 min. Then, the section was retrieved by EDTA. The primary antibodies were as follows: anti-ZEB1 (1 : 4000 dilution, Cat. 21544-1-AP, ProteinTech) and anti-PD-L1 (Ready-to-use, Cat. GT2280, GeneTech). The immunostained section was scanned using Aperio Digital Pathology Slide Scanner. The semiquantitative assessment was conducted according to the 12-point criteria as described previously, and the semiquantitative result was defined as immunoreactivity score (IRS) [19].

### 2.5. Cell Culture and Transfection

Human CRC cell lines HCT116 and SW620 were purchased from KeyGEN (Nanjing, China). HCT116 cells were maintained in McCoy's 5A media supplemented with 10% fetal bovine serum (FBS) at 37°C with 5% CO_2_. SW620 cells were cultured in Leibovitz's L-15 media supplemented with 10% FBS at 37°C with 5% CO_2_. All experiments were performed with mycoplasma-free cells. The HCT116 and SW620 cell lines have recently been authenticated using short tandem repeat profiling.

For knockdown or upregulation of ZEB1 expression, CRC cells were cultured in 6-well plates to 60–80% confluence and transfected with small interfering RNA (siRNA) and overexpression vector for ZEB1, which is synthesized in KeyGEN, using Lipofectamine 3000 Reagent (Cat. L3000015, Invitrogen, CA, USA) according to the manufacturer's instructions. The sequence of ZEB1 siRNAs were as follows: siRNA-1: AGGAAGAGGAGGAGGAUAATT, siRNA-2: ACACAUAAGCAGUAAGAAATT, and siRNA-3: GGCAAAAGAUAGAGAAUAATT. After 48 hours, the total RNA and protein of CRC cells were extracted and submitted for quantitative real-time PCR (qRT-PCR) and Western blotting analysis to check the efficiency of ZEB1 knockdown and overexpression.

### 2.6. Quantitative Real-Time PCR

The total RNA of HCT116 and SW620 cells was collected by TRIzol reagent (Cat. 15596026, Invitrogen, CA, US). The primers for ZEB1, PD-L1, and GAPDH mRNA reverse transcription were synthesized in KeyGEN (Nanjing, China). qRT-PCR was conducted using the One-Step TB GreenTM PrimeScriptTM RT-PCR Kit II (SYBR Green) (Cat. RR086 B, TaKaRa, Kyoto, Japan).

Primers used for gene amplification were as follows: ZEB1: (forward) CGCTTCTCACACTCTGGGTCTT and (reverse) CCTCTTCCTGCTCTGTGCTGTC; PD-L1: (forward) GCCGAAGTCATCTGGACAAGC and (reverse) TGATTCTCAGTGTGCTGGTCAC; GAPDH: (forward) AGATCATCAGCAATGCCTCCT and (reverse) TGAGTCCTTCCACGATACCAA.

### 2.7. Western Blotting Analysis

CRC cells were plated in a 6-well plate for transfection. After 48 hours, the total protein of HCT116 and SW620 cells was harvested using lysis buffer. Then, SDS-PAGE and Western blotting analyses were conducted as standard protocols. The primary antibodies used were as follows: ZEB1 (1 : 1000 dilution, Cat. 21544-1-AP, ProteinTech), E-cadherin (1 : 1000 dilution, Cat. 20874-1-AP, ProteinTech), N-cadherin (1 : 1000 dilution, Cat. 22018-1-AP, ProteinTech), PD-L1 (1 : 2000 dilution, Cat. 66248-1-Ig, ProteinTech), and GAPDH (1 : 5000 dilution, Cat. 60004-1-Ig, ProteinTech). Expression levels of these proteins were standardized to GAPDH for each specimen.

### 2.8. Immunofluorescence

The subcellular locations of proteins were assessed using immunofluorescence assay according to standardized protocols [20]. The primary antibodies used were as follows: ZEB1 (1 : 100 dilution, Cat. 21544-1-AP, ProteinTech), E-cadherin (1 : 100 dilution, Cat. 20874-1-AP, ProteinTech), N-cadherin (1 : 100 dilution, Cat. 22018-1-AP, ProteinTech), and PD-L1 (1 : 100 dilution, Cat. 66248-1-Ig, ProteinTech). The stained cells were observed under a fluorescence microscope (FV3000, Olympus).

### 2.9. CCK-8 Assay

HCT116 and SW620 cells were digested using 0.25% trypsin for 1 min and resuspended with DMEM media containing 10% FBS. Suspended cells were seeded on a 96-well plate with the cell density adjusted to 5 × 10^4^ cells/ml (100 *μ*l/well) and fostered at 37°C in a constant-temperature incubator with 5% CO_2_ for 48 hours. To each well, 10 *μ*l CCK-8 was added, after which the plate was put in the incubator for 1 hour. The OD value of each well was measured at 450 nm by a microplate reader.

### 2.10. Wound Healing Assay

For cell migration assays, HCT116 and SW620 cells were seeded in 6-well plates (Costar, Corning, NY) and cultured to 80% confluence. The monolayers of cells were wounded by removing the culture-insert and rinsed with PBS to remove cell debris. After the 24 h migration, cells were stained with 0.2% crystal violet for 20 min at room temperature. The images were acquired at times 0 h and 24 h after migration using a microscope (magnification: 100×, OLYMPUS IX51). The migratory distance was calculated by the minus of the edge of the wound closure between 0 h and 24 h.

### 2.11. Boyden Chamber Assay

For cell invasion assays, 1 × 10^5^ cells in serum-free medium supplemented with 5 mg/mL BSA were inoculated to the upper sides of the modified Boyden chamber (8.0-*μ*m, Cat. 3422, Corning, NY, US). The polycarbonate membranes of Boyden chambers were coated with Matrigel (BD Biosciences, NJ, US). After 24 hours, the invasive cells on the lower sides of Boyden chambers were fixed and stained with 0.2% crystal violet for 20 min at room temperature. The pictures of stained cells were captured by a microscope (magnification: 200×, OLYMPUS IX51).

### 2.12. In Vivo Tumorigenesis Analysis

C57BL/6 mice (4-5 weeks old) were purchased from Shanghai SLAC Laboratory Animal Co., Ltd. The mice were raised in SPF-grade experimental animal centers and provided with free access to food and water. To establish the syngeneic mouse model, PD-L1 positive MC38 mouse tumor cells [21, 22] maintained in DMEM media supplemented with 10% FBS were subcutaneously injected into the flanks of these female mice (1 × 10^7^ cells). Tumors were monitored and regularly measured with calipers every two to three days. When tumors reached about 100 mm^3^ in volume, mice were randomized into two different groups (*n* = 6): vehicle group and Biochanin A-treated group. Biochanin A was dissolved in PBS and administered to mice through intraperitoneal injection at 50 mg/kg daily. On day 21, the mice were anesthetized with a 0.5% sodium pentobarbital solution to remove the tumor and photographed and weighted, and the tissues were submitted for IHC staining. All experiments were approved by the Laboratory Animal Ethics Committee at the Affiliated Wuxi People's Hospital of Nanjing Medical University. Mouse tumor tissues were manufactured into 4 mm thick formalin-fixed and paraffin-embedded sections. IHC for these sections was subsequently conducted. The primary antibodies used in the research were as follows: anti-ZEB1 (1 : 500 dilution, Cat. 21544-1-AP, ProteinTech) and anti-PD-L1 (1 : 100 dilution, Cat. 66248-1-Ig, ProteinTech). Semiquantitative evaluation criteria were as described previously.

### 2.13. Statistical Analysis

All statistical analyses were conducted using SPSS 26.0 software (Chicago, IL). Most of the data were analyzed by Student's *t*-test or Mann–Whitney test. All data are presented as means ± SDs. Correlation analysis between two variables was examined by Pearson's test. Two-sided *P* value ≤ 0.05 was considered statistically significant labeled with ^*∗*^*p* < 0.05; ^*∗∗*^*p* < 0.01; ^*∗∗∗*^*p* < 0.01.

## 3. Results

### 3.1. EMT Was Associated with Features of TME in CRC

**Figure 1:**
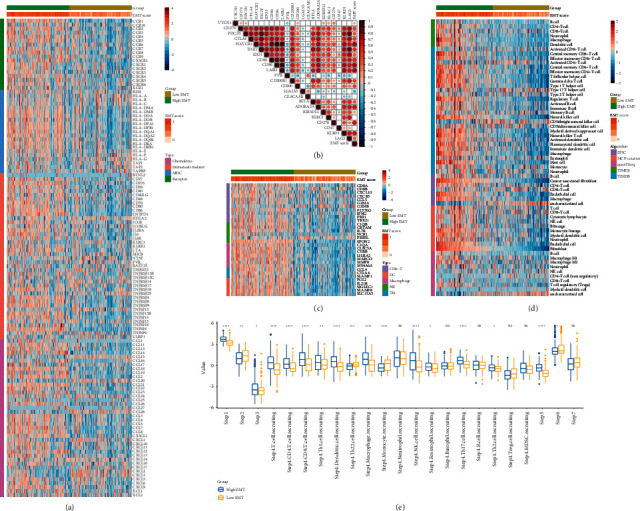
EMT was related to the features of TME in CRC. (a) Expression levels of 122 immunomodulators between the high and low EMT groups. (b) Correlation between EMT score and inhibitory immune checkpoints. The color reveals the Pearson correlation coefficient. (c) Expression levels of gene markers of immune cells between the high and low EMT groups. (d) The difference in TIICs levels between high and low EMT groups calculated by five algorithms. (e) Differences in the various steps of the cancer immunity cycle between the high and low EMT groups.

Previous research revealed that EMT was correlated with PD-L1 expression in clinical lung cancer cohorts [23]. Thus, we first investigated the immunological role of EMT in CRC in this research. A majority of immunostimulators, major histocompatibility complex (MHC) molecules, chemokines, and chemokine receptors were highly expressed in the high EMT group (Figure 1(a)). Tumor Purity, ESTIMATE Score, Immune Score, and Stromal Score were calculated by the ESTIMATE method. The results showed that the EMT score was positively correlated with ESTIMATE Score, Immune Score, and Stromal Score but negatively correlated with Tumor Purity (Figures S1(a)–S1(d)). EMT score was revealed to be positively related to a majority of immune checkpoints, including PD-L1, PD-1, and CTLA-4 (Figure 1(b)). In addition, the EMT score was positively related to most gene markers of immune cells (Figure 1(c)). Next, we also estimated the infiltration level of TIICs based on five independent algorithms. EMT score was positively related to the infiltration levels of most immune cells (Figure 1(d)). Furthermore, the activities of most steps in antitumor immunity cycles were enhanced in the high EMT group (Figure 1(e)). Overall, these findings revealed that EMT was significantly related to the inflammatory TME in CRC.

### 3.2. ZEB1 Was Positively Related to and Regulated PD-L1 Expression in CRC

**Figure 2:**
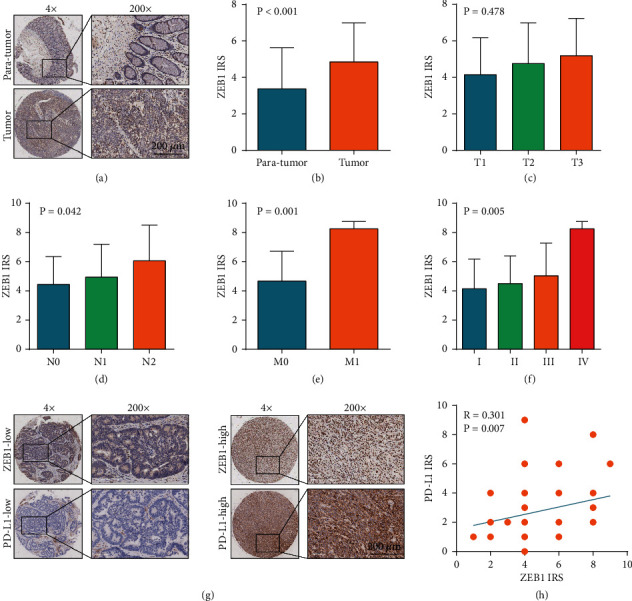
Expression of ZEB1 in CRC and its correlation with PD-L1 expression. (a) Representative images uncovering ZEB1 expression in paratumor and tumor tissues using anti-ZEB1 staining. (b) Semiquantitative analysis of ZEB1 in paratumor and tumor tissues. (c) Semiquantitative analysis of ZEB1 in CRC tissues with different T stages. (d) Semiquantitative analysis of ZEB1 in CRC tissues with different N stages. (e) Semiquantitative analysis of ZEB1 in CRC tissues with different M stages. (f) Semiquantitative analysis of ZEB1 in CRC tissues with different TNM clinical stages. (g) Representative images uncovering low and high ZEB1 and PD-L1 expressions in CRC tissues using anti-ZEB1 and anti-PD-L1 staining. (h) Correlation between ZEB1 and PD-L1 expressions in CRC tissues.

**Figure 3:**
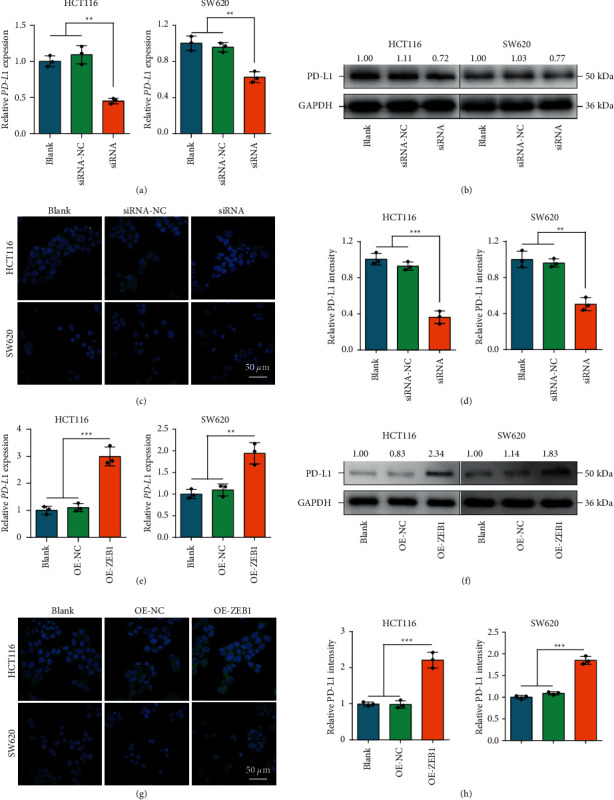
The regulatory effect of ZEB1 on PD-L1 expression in CRC. (A, B) Transcriptional and protein expressions of ZEB1 in CRC cells with ZEB1 silencing were examined by qPCR and Western blotting. (C, D) Protein expression of PD-L1 in CRC cells with ZEB1 silencing was examined by immunofluorescence. (E, F) Transcriptional and protein expressions of ZEB1 in CRC cells with ZEB1 overexpression were examined by qPCR and Western blotting. (G, H) Protein expression of PD-L1 in CRC cells with ZEB1 overexpression was examined by immunofluorescence.

Among the regulators of the EMT process, ZEB1 has been proved to be a critical regulator for PD-L1 expression in lung cancer [23], breast cancer [24], gastric cancer [25], and diffuse large B cell lymphoma [26]. Thus, we investigated the correlation between ZEB1 and PD-L1 in CRC. First, ZEB1 was upregulated in CRC tissues compared with paratumor tissues (Figures 2(a) and 2(b)) and positively correlated with tumor stages, including N stage, *M* stage, and TNM clinical stage (Figures 2(c)–2(f)). In addition, ZEB1 was positively correlated with PD-L1 expression revealed by IHC staining (Figures 2(g)–2(h)). Next, we investigated whether ZEB1 regulated PD-L1 expression in CRC cells. The efficiency of siRNAs for ZEB1 silencing was checked, and the results showed that siRNA-2 (ACACAUAAGCAGUAAGAAATT) had a satisfactory silencing efficiency in both HCT-116 and SW620 cells (Figures S2(a) and S2(b)). In addition, the overexpression efficiency of the ZEB1 vector was also validated (Figures S2(c)–S2(d)). Inhibition of ZEB1 significantly downregulated PD-L1 expression (Figures 3(a)–3(d)), but ZEB1 overexpression notably upregulated PD-L1 in both HCT-116 and SW620 cells (Figures 3(e)–3(h)). Taken together, these findings suggested that ZEB1 might be a critical regulator for PD-L1 expression in CRC as well.

### 3.3. Biochanin A Suppressed ZEB1 Expression and the EMT Process in CRC Cells

**Figure 4:**
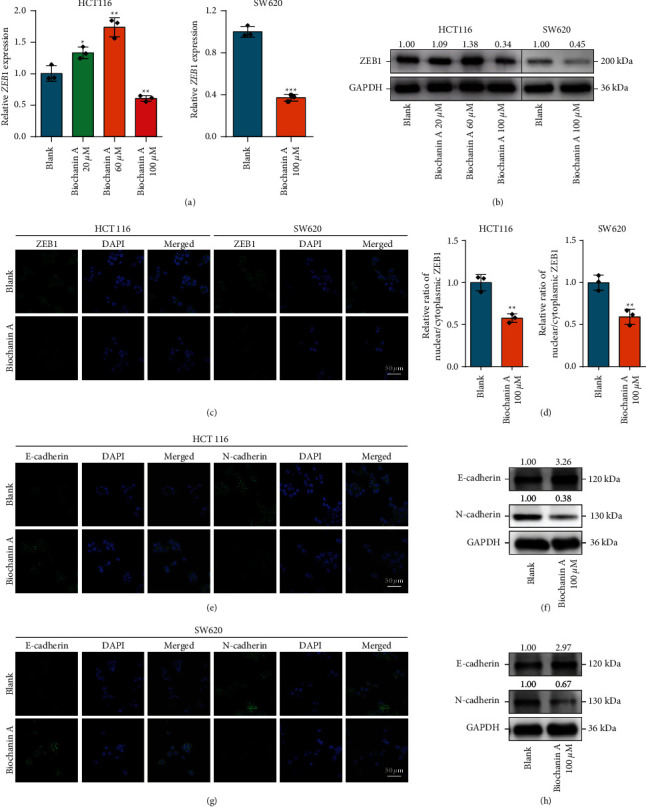
The effect of Biochanin A on ZEB1 expression and EMT in CRC. (A, B) Transcriptional and protein expressions of ZEB1 in CRC cells treated with Biochanin A were examined by qPCR and Western blotting. (C, D) Protein expression of ZEB1 in CRC cells treated with Biochanin A was examined by immunofluorescence. (E, G) Protein expression of E-cadherin and N-cadherin in CRC cells treated with Biochanin A was examined by immunofluorescence. (F, H) Protein expression of E-cadherin and N-cadherin in CRC cells treated with Biochanin A was examined by Western blotting.

Biochanin A was reported as an inhibitor for the EMT process [27]. Therefore, we checked the pharmacological effects of Biochanin A on ZEB1 expression and the EMT process in CRC cells. Low doses (20 *μ*M and 60 *μ*M) of Biochanin A slightly upregulated but a high dose (100 *μ*M) significantly inhibited ZEB1 expression in HCT116 cells (Figures 4(a) and 4(b)). In addition, a high dose of Biochanin A also downregulated ZEB1 expression in SW620 cells (Figures 4(a) and 4(b)). Next, immunofluorescence assay also proved that a high dose of Biochanin A downregulated ZEB1 expression, especially nuclear ZEB1 expression in CRC cells (Figures 4(c) and 4(d)). We also checked the effects of Biochanin A on the EMT process. The results exhibited that Biochanin A transformed CRC cells into epithelial phenotype and upregulated E-cadherin but inhibited N-cadherin expression in CRC cells (Figures 4(e)–4(h)). Collectively, all evidence supported that Biochanin A inhibited ZEB1 expression and blocked the EMT process in CRC.

### 3.4. Biochanin A Suppressed ZEB1-Mediated Aggressiveness of CRC Cells

**Figure 5:**
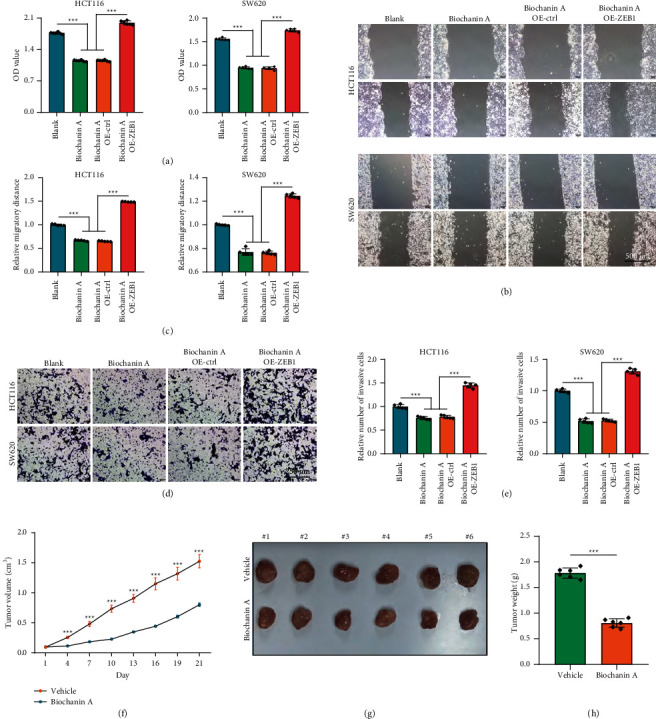
Tumor-suppressive role of Biochanin A in CRC in vitro and in vivo. (a) The proliferative capacity of control and Biochanin A-treated CRC cells was examined by CCK-8 assay. (b) The migratory capacity of control and Biochanin A-treated CRC cells was examined by wound healing assay. (c) Quantitative analysis of cell migratory capacity. (d) The invasive capacity of control and Biochanin A-treated CRC cells was examined by Boyden chamber assay. (e) Quantitative analysis of cell invasive capacity. (f) Tumor volume of the mouse models, which was monitored every three days. (g) Isolated tumor images from these two groups on day 21 after the first treatment. (h) The tumor weight of these two groups; mice were sacrificed on day 21 after the first treatment.

We next assessed the tumor-suppressive role of Biochanin A in CRC cells. Compared with the control cells, Biochanin A-treated HCT-116 and SW620 cells exhibited attenuated proliferative capacity (Figure 5(a)). In addition, Biochanin A treatment significantly inhibited the migratory and invasive capacities of CRC cells (Figures 5(b)–5(e)). However, ZEB1 overexpression rescued Biochanin A-induced inhibition of proliferation, migration, and invasion (Figures 5(a)–5(e)). Moreover, the antitumor pharmacodynamic of Biochanin A in vivo was also evaluated. The results showed that Biochanin A notably inhibited tumor growth of MC-38 mouse CRC cells in vivo (Figures 5(f)–5(h)). Overall, Biochanin A significantly suppressed CRC progression in vitro and in vivo via downregulating ZEB1 expression, which could be used as a novel antitumor drug.

### 3.5. Biochanin A Suppressed ZEB1-Mediated PD-L1 Expression

**Figure 6:**
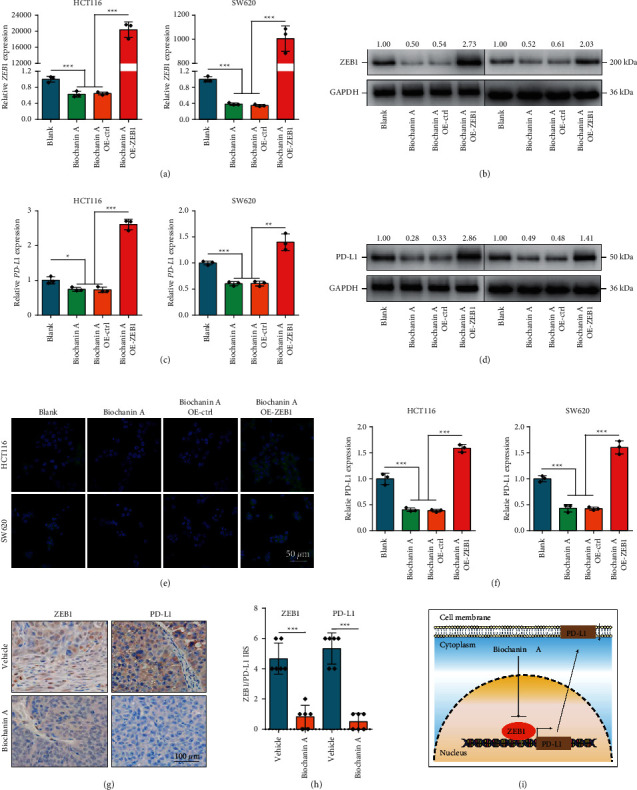
The effect of Biochanin A on PD-L1 expression in CRC. (A, B) Transcriptional and protein expressions of ZEB1 in CRC cells treated with Biochanin A or transfected with ZEB1 vector were examined by qPCR and Western blotting. (C, D) Transcriptional and protein expressions of PD-L1 in CRC cells treated with Biochanin A or transfected with ZEB1 vector were examined by qPCR and Western blotting. (E, F) Protein expression of PD-L1 in CRC cells treated with Biochanin A or transfected with ZEB1 vector was examined by immunofluorescence. (G) Representative images uncovering ZEB1 and PD-L1 expression in vehicle and Biochanin A treated groups. (H) Semiquantitative analysis of ZEB1 and PD-L1 in mouse tumor tissues. (I) Schematic diagram of the mechanism underlying Biochanin A-induced ZEB1-mediated PD-L1 downregulation.

Given the crucial regulatory role of ZEB1 in PD-L1 expression, we also checked the effect of Biochanin A on PD-L1 expression. Predictably, Biochanin A inhibited ZEB1 and PD-L1 overexpression, and overexpression of ZEB1 largely rescued Biochanin A-induced PD-L1 downregulation (Figures 6(a)–6(d)). In addition, the result was also validated by immunofluorescence assay (Figures 6(e)–6(f)). Furthermore, IHC analysis of mouse tumor tissues revealed that Biochanin A suppressed ZEB1 and PD-L1 expression in vivo as well (Figures 6(g) and 6(h)). To sum up, Biochanin A was an antitumor immunomodulator via inhibiting ZEB1-mediated PD-L1 expression and thus increased the immune cell infiltration (Figure 6(i)).

## 4. Discussion

As one of the significant features of malignant tumors, immune escape plays a significant role in the oncogenesis, progression, and therapeutic resistance of malignant tumors [28]. Immunotherapeutic drugs represented by PD-1/PD-L1 inhibitors have been widely used in multiple malignant diseases [29–32]. PD-L1 could inhibit the proliferation of T lymphocytes by binding to PD-1 and reduce the activity of T lymphocytes, thereby negatively regulating the antitumor immune response [33, 34]. Although the clinical application of PD-1/PD-L1 inhibitors is limited in CRC, the role of PD-L1 in promoting progression and immune evasion of CRC could not be ignored [35]. Downregulation of PD-L1 expression or blocking PD-L1 signals could block progression and immune evasion of CRC. For instance, PPAR*γ* agonists could increase the expression of PD-L1 at transcriptional and protein levels in CRC cell lines and induce immune evasion [36]. Given the complexity and diversity of tumor cell gene expression regulation, it is indispensable to further investigate the molecular mechanisms underlying PD-L1 regulation and find new intervention targets.

In this research, we reported that EMT was essential for PD-L1 expression. Based on the bioinformatics analysis, we found that the EMT score was positively related to the expression of the most significant immunomodulators. EMT score was also positively related to increased infiltration of TIICs and hyperactive cancer immunity cycles. In addition, we revealed that a high EMT score was positively correlated with most immune checkpoints expression and negatively correlated with Tumor Purity. Previous research indicated that ZEB1 was the critical regulator for PD-L1 expression among a panel of transcription factors in the EMT process in breast cancer [24]. Similarly, we validated that ZEB1 positively regulated PD-L1 expression in CRC. In addition, the positive correlation between ZEB1 and PD-L1 in clinical samples was also observed.

In the past few decades, an increasing number of traditional Chinese medicines have been reported to exert tumor-suppressive functions, which may open up novel insights for tumor therapy [37, 38]. Biochanin A is an oxymethylated isoflavone compound, which is widely present in some edible plants. Existing studies have shown that Biochanin A has a variety of pharmacological effects, such as antitumor, anti-inflammatory, antibacterial, hypoglycemic, antioxidant, and neuroprotection [39]. Biochanin A could induce S phase arrest and apoptosis of lung cancer cells [40]. In addition, Biochanin A negatively regulated the proliferation and migration of lung cancer cells by inhibiting the VEGF/VEGFR2 signaling pathway [41]. Furthermore, the antitumor effect of Biochanin A was also confirmed in pharyngeal squamous cell carcinoma [42] and breast cancer [43]. All the evidence suggested that Biochanin A was a broad-spectrum antitumor candidate. In this research, we found that Biochanin A significantly downregulated ZEB1 expression and blocked the EMT process. Moreover, Biochanin A inhibited proliferation, migration, and invasion of HCT116 and SW620 cells, which could be rescued by ZEB1 overexpression. Given the critical role of ZEB1 in regulating PD-L1 expression, we speculated that Biochanin A may downregulate PD-L1 via inhibiting ZEB1 expression. Notably, Biochanin A indeed suppressed PD-L1 expression, which was rescued by ZEB1 overexpression.

## 5. Conclusion

In summary, we reported that the EMT process is related to features of TME in CRC in this research. Besides, ZEB1 is a crucial regulator for PD-L1 expression, and pharmacological inhibition of ZEB1 using Biochanin A downregulated PD-L1 in vivo. Overall, we uncovered that Biochanin A is a promising antitumor candidate by negatively regulating the EMT process and PD-L1 expression.

## Data Availability

The data of this research are available from the corresponding author upon request.

## Supplementary Materials

Supplementary Materials Figure S1: Correlations between EMT score and scores estimated by ESTIMATE algorithm. (a) Positive correlation between EMT score and immune score. (b) Positive correlation between EMT score and ESTIMATE Score. (c) Positive correlation between EMT score and Stromal Score. (d) Negative correlation between EMT score and Tumor Purity. Figure S2: The efficiency of ZEB1 silencing and overexpression in CRC cells. (a, b) The efficiency of ZEB1 silencing in CRC cells was assessed by qRT-PCR and Western blotting. (c, d) The efficiency of ZEB1 overexpression in CRC cells was assessed by qRT-PCR and Western blotting.
