# Supplementary material for: Biochanin A Suppresses Tumor Progression and PD-L1 Expression via Inhibiting ZEB1 Expression in Colorectal Cancer
Source: J Oncol. 2022 Feb 22;2022:3224373. doi: 10.1155/2022/3224373 (PMC8888121; doi:10.1155/2022/3224373)
Supplement: Supplementary Materials — Figure S1: Correlations between EMT score and scores estimated by ESTIMATE algorithm. (a) Positive correlation between EMT score and immune score. (b) Positive correlation between EMT score and ESTIMATE Score. (c) Positive correlation between EMT score and Stromal Score. (d) Negative correlation between EMT score and Tumor Purity. Figure S2: The efficiency of ZEB1 silencing and overexpression in CRC cells. (a, b) The efficiency of ZEB1 silencing in CRC cells was assessed by qRT-PCR and Western blotting. (c, d) The efficiency of ZEB1 overexpression in CRC cells was assessed by qRT-PCR and Western blotting. [file 3224373.f1.docx]

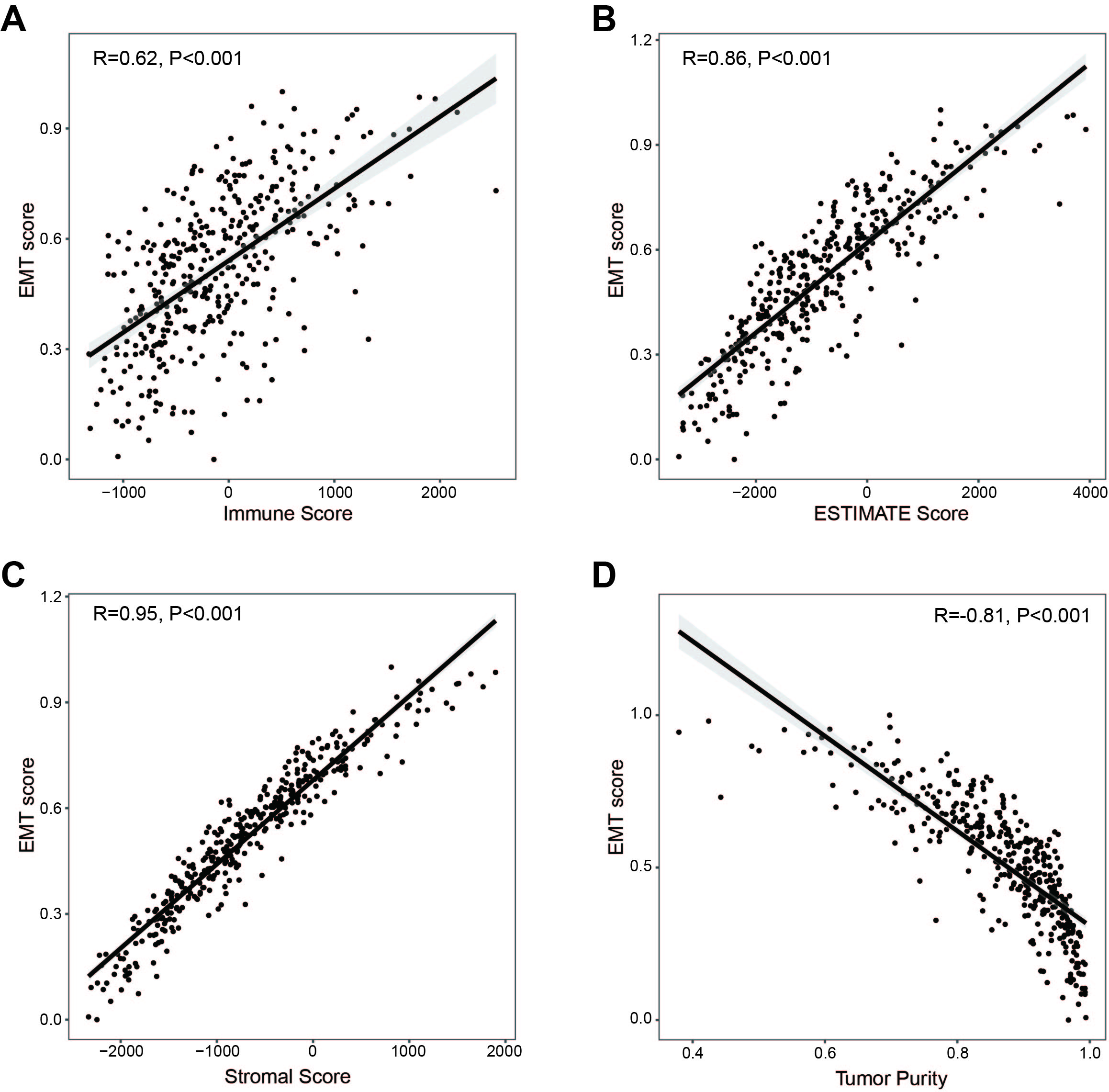


**Figure S1. Correlations between EMT score and scores estimated by ESTIMATE algorithm.**

(A) Positive correlation between EMT score and immune score. (B) Positive correlation between EMT score and ESTIMATE score. (C) Positive correlation between EMT score and stromal score. (D) Negative correlation between EMT score and tumor purity.

**
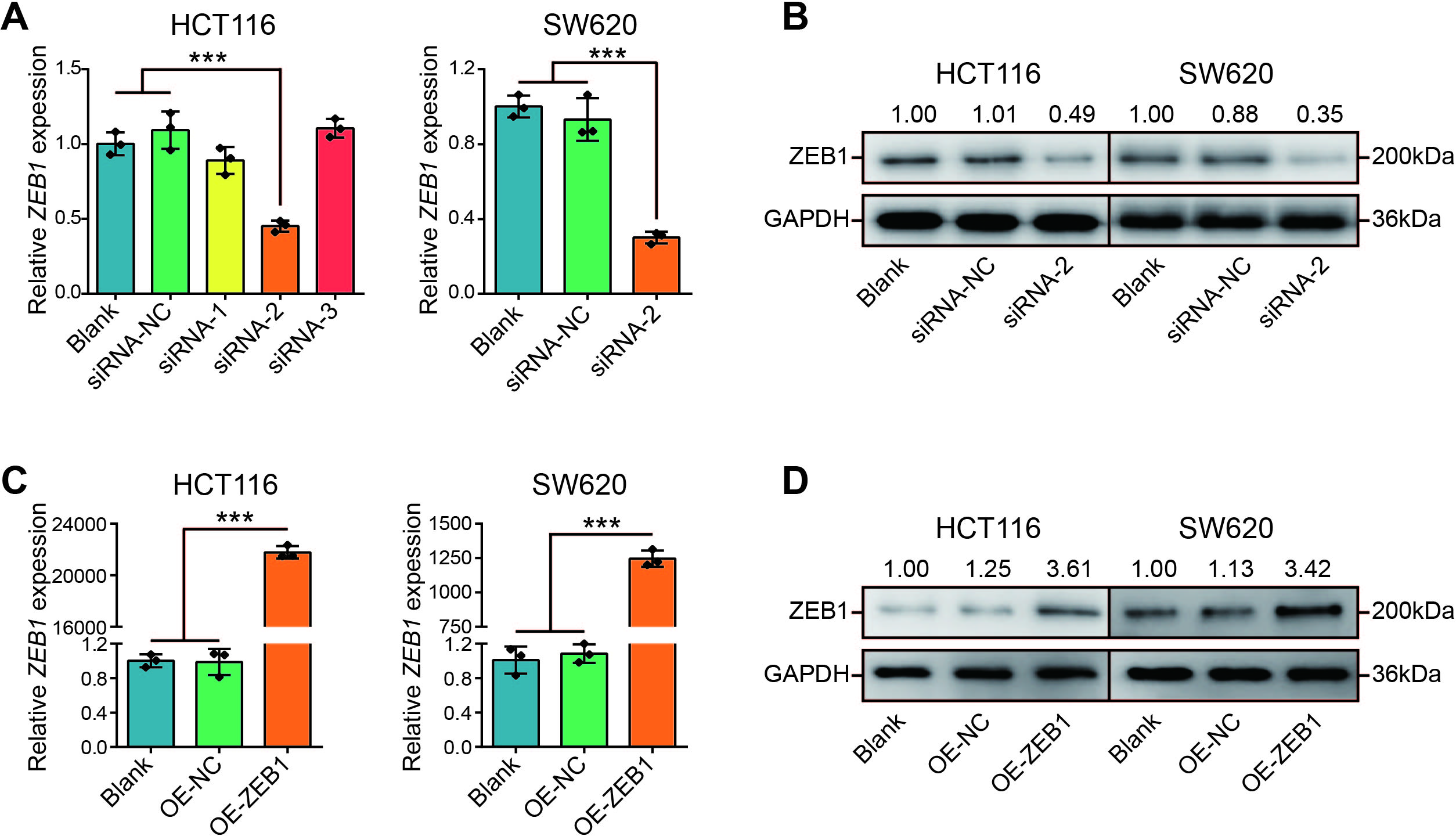
**

**Figure S2. The efficiency of ZEB1 silencing and overexpression in CRC cells.**

(A, B) The efficiency of ZEB1 silencing in CRC cells was assessed by qRT-PCR and Western blotting. (C, D) The efficiency of ZEB1 overexpression in CRC cells was assessed by qRT-PCR and Western blotting.
